# Use of combined laboratory and ultrasonography results to select patients for biliary tract imaging or intervention: a retrospective cohort study 

**Published:** 2019

**Authors:** Shahab Hajibandeh, Shahin Hajibandeh, Joseph Thompson, Jonaid Mohammed, Christopher Smith, James Prince, Charlotte Lisberg, Leo Watton, Nathan Peter, Whajong Lee, Vivek Trivedi, Nicholas Hobbs, Jigar Shah, Rao Muhammad Asaf Khan, Sanjay Dalmia, Sohail Malik, Moustafa Mansour

**Affiliations:** 1 *Department of General Surgery, North Manchester General Hospital, Manchester, UK *; 2 *Department of General Surgery, Sandwell and West Birmingham Hospitals NHS Trust, Birmingham, UK*; © **equally contributed to this paper**

**Keywords:** Liver function test, Biliary calculi, Common bile duct, Magnetic resonance cholangiopancreatography

## Abstract

**Aim:**

To determine whether combined laboratory and ultrasonography results can be used to select patients for biliary tract imaging (BTI) or intervention.

**Background:**

Despite ongoing research, selection of patients with suspected CBD stone (CBDS) for BTI or direct intervention without imaging is still a subject of debate.

**Methods:**

Patients aged≥18 with symptomatic gallstone disease (SGD) who underwent MRCP over 3 years (2014-2017) were divided into the following cohorts: Group A: Normal liver enzymes with normal CBD diameter; Group B: Normal liver enzymes with dilated CBD; Group C: Isolated rise of liver enzymes with normal CBD diameter; Group D: Isolated rise of liver enzymes with dilated CBD; Group E: Hyperbilirubinemia with normal CBD diameter; Group F: Hyperbilirubinemia with dilated CBD. Binary logistic regression models were constructed for analyses.

**Results:**

Overall, 1022 patients were included. The frequency of CBDS was 7.2% in Group A; 3.8% in Group B; 6.3% in Group C; 22% in Group D; 24.2% in Group E; 47.4% in Group F. Hyperbilirubinemia with normal CBD (OR:1.52,P=0.010) and hyperbilirubinemia with dilated CBD (OR:5.12,P<0.001) independently predicted CBDS. Normal or isolated rise of liver enzymes with or without dilated CBD did not predict CBDS. Combined laboratory and ultrasonography had positive predictive value and negative predictive value of up to 47.37% and 100%, respectively.

**Conclusion:**

Patients with isolated rise of liver enzymes or hyperbilirubinemia with or without dilated CBD should undergo BTI prior to ERCP. Direct ERCP could be preserved for patients with high suspicion of CBDS where clinical features do not allow waiting for BTI.

## Introduction

 Choledocholithiasis or stones in the common bile duct (CBD) mainly result from the passage of gallstones through the cystic duct into the CBD but they may be formed in the CBD itself. CBD stones are associated with morbidity and even mortality as they can cause biliary obstruction, cholangitis, pancreatitis, or secondary biliary cirrhosis in patients who have had the stones for a long time (1). The management of patients with gallstone disease suspected of having CBD stones includes evaluation of the probability of stones in the CBD, treatment of these stones when present and treatment of the stones in the gallbladder (1). 

A number of techniques including magnetic resonance cholangiopancreatography (MRCP), endoscopic retrograde cholangiopancreatography (ERCP) or endoscopic ultrasonography (EUS) have been suggested as methods to predict the presence of CBD stones preoperatively. Magnetic resonance cholangiopancreatography (MRCP) is a safe, non-invasive means of imaging the biliary tree with good accuracy for detecting CBD stones (2). It is normally considered when ultrasound (US) scan has not detected CBD stones but the CBD is dilated or liver function test results (LFTs) are abnormal. MRCP might both avoid the need for intraoperative CBD imaging and reduce the unnecessary endoscopic sphincterotomy rate (2,3). 

In spite of the advantages, it has been argued that the use of MRCP for universal preoperative duct interrogation or for imaging in patients with suspicion of CBD stone is not cost-effective since a significant proportion of patients undergoing MRCP do not have CBD stones or might have passed stones (4). Therefore, appropriate patient selection for MRCP is deemed important. Mercer et al. (5) suggested that patients with normal LFTs and CBD diameter require no further imaging; those with a high suspicion of CBD stones should progress directly to ERCP; patients should undergo further investigation using MRCP or EUS where the CBD status is uncertain. If MRCP or EUS demonstrates CBD stones, patients should undergo ERCP with sphincterotomy, while those with a clear CBD require no further investigation (5). 

We aimed to determine whether combined LFTs and US scan results can be used to identify a subgroup of patients with suspected CBD stone who should be selected for biliary tract imaging and those should be selected for direct ERCP. 

## Methods

Upon gaining approval from our local Clinical Governance and Audit Unit, we conducted a retrospective study at our Upper Gastrointestinal Surgery Unit (UGISU) to determine whether combined LFTs and US scan results can be used to identify a subgroup of patients with suspected CBD stone who should be selected for biliary tract imaging and those should be selected for direct ERCP.

We searched the Picture Archiving and Communications System (PACS) to identify all patients who underwent MRCP for suspected CBD stones between January 2014 and January 2017. We included patients aged ≥18 with available LFTs and US scan results who underwent MRCP within 28 days of admission solely for suspected CBD stone. We excluded patients who were known to have CBD stone(s); patients who had previous cholecystectomy; patients who underwent MRCP for suspected primary or secondary sclerosing cholangitis, cholangiocarcinoma or CBD stricture. 

The eligible patients were divided into following 6 groups based on their LFTs and US scan results: 1) Normal liver enzymes with normal CBD diameter (Group A), 2) Normal liver enzymes with dilated CBD (Group B), 3) Isolated rise of liver enzymes with normal CBD diameter (Group C), 4) Isolated rise of liver enzymes with dilated CBD (Group D), 5) Hyperbilirubinemia with normal CBD diameter (Group E), and 6) Hyperbilirubinemia with dilated CBD (Group F). Isolated rise of liver enzymes as raised alkaline phosphatase (ALP) and/or alanine transaminase (ALT) but normal bilirubin. Hyperbilirubinemia was defined as raised bilirubin with raised or normal ALP and/or ALT. The extrahepatic biliary tree diameter of greater than 7mm on US scan was defined as dilated CBD. 

**Table 1:** Baseline characteristics of the included population

	All patients	Normal liver enzymes + Normal CBD	Normal liver enzymes + Dilated CBD	Isolated rise of liver enzymes + Normal CBD	Isolated rise of liver enzymes + Dilated CBD	Hyperbilirubinemia + Normal CBD	Hyperbilirubinemia + Dilated CBD
Age	58.6±0.59*	53.5±1.59	56.9±1.75	56.8±1.32	55.7±2.05	61.3±0.96	62.3±1.62
Male	344/1022 (33.7)†	28/138 (20.3)	10/106 (9.4)	74/190 (38.9)	26/90 (28.9)	152/346 (43.9)	54/152 (35.5)
Female	678/1022(66.3)	110/138(79.7)	96/106 (90.6)	116/190 (61.1)	64/90 (71.1)	194/346 (56.1)	98/152 (64.5)
Bilirubin	32.5±1.06	12.6±1.52	10.1±0.51	12.7±0.34	11.4±0.45	54.8±2.08	52.6±2.60
ALP	199.2±5.19	87.3±2.31	93.5±2.82	183.6±8.69	183.5±16.4	262.6±11.1	258.8±12.2
ALT	180.8±8.74	23.9±2.99	19.6±0.71	129.1±7.53	96.6±10.3	305.1±21.3	267.1±19.4
CBD diameter on US	7.2±0.09	5.7±0.09	10.3±0.24	5.7±0.06	9.3±0.20	5.69±0.05	10.5±0.24
Gallbladder sludge on US	154/1022 (15.1)	16/138 (11.6)	12/106 (11.3)	30/190(15.8)	14/90 (15.6)	58/346 (16.8)	24/152 (15.8)
Cholecystitis on US	299/1022 (29.3)	16/138 (11.6)	28/106 (26.4)	60/190 (31.6)	32/90(35.6)	110/346 (31.8)	53/152 (34.9)

LFT: liver function tests; CBD: common bile duct; US: ultrasound; MRCP: magnetic resonance cholangiopancreatography

* mean±SD;

† n/total (Percent);

**Table 2:** Frequency of CBD stone in different subgroups

	All patients	Normal liver enzymes +Normal CBD	Normal liver enzymes +Dilated CBD	Isolated rise of liver enzymes +Normal CBD	Isolated rise of liver enzymes +Dilated CBD	Hyperbilirubinemia +Normal CBD	Hyperbilirubinemia +Dilated CBD
All patients	202/1022 19.7 (17.4-22.4)*	10/138 7.2 (3.7%-13.3%)	4/106 3.8 (1.2-9.9)	12/190 6.3 (3.4 -11.0)	20/90 22 (14.4-32.4)	84/346 24.2 (19.9-29.2)	72/152 47.4(39.3-55.6)
Age <55	54/408 13.2 (10.2-17.0)	0/64 0	2/50 4 (0.7 -14.9)	6/86 6.9 (2.8-15.1)	8/40 20 (9.6-36.1)	18/120 15 (9.4-22.9)	20/48 41.7(27.9-56.7)
Age ≥55	148/614 24.1 (20.8-27.7)	10/74 13.5 (7.0-23.9)	2/56 3.6 (0.6 -13.4)	6/104 5.8 (2.4-12.6)	12/50 24 (13.5-38.4)	66/226 29.2 (23.5-35.7)	52/104 50(40.1-59.9)
No Cholecystitis	127/723 17.6 (14.9 -20.6)	8/122 6.6 (3.1-12.9)	0/78 0	6/130 4.6 (1.9-10.2)	16/58 27.6 (17.1-41.1)	60/236 25.4(20.1-31.6)	37/99 37.4(28.0-47.7)
Cholecystitis	75/299 25.1 (20.4-30.5)	2/16 12.5 (2.2-39.6)	4/28 14.3 (4.7-33.6)	6/60 10 (4.1-21.2)	4/32 12.5 (4.1-29.9)	24/110 21.9(14.7-30.9)	35/53 66.0 (51.6-78.1)

LFT: liver function tests; CBD: common bile duct; US: ultrasound; MRCP: magnetic resonance cholangiopancreatography; Values in parentheses: 95% confidence intervals

* Percent (95% confidence interval)

**Table 3:** Results of logistic binary regression analysis of individual baseline variables (independent variable) and CBD stone (dependent variable)

	All patients		Age <55		Age ≥55	
OR	P-value	OR	P-value	OR	P-value
Age	1.0251 (1.0160, 1.0344)	<0.001	1.0350 (1.0041, 1.0668)	0.023	1.0270 (1.0083, 1.0460)	0.004
Male	1.4553 (1.0601, 1.9979)	0.021	1.2385 (0.6411, 2.3925)	0.53	1.3027 (0.8973, 1.8914)	0.165
Bilirubin	1.0131 (1.0090, 1.0172)	<0.001	1.0071 (1.0002, 1.0141)	0.05	1.0159 (1.0106, 1.0213)	<0.001
ALP	1.0037 (1.0028, 1.0047)	<0.001	1.0055 (1.0035, 1.0076)	<0.001	1.0028 (1.0017, 1.0038	<0.001
ALT	1.0009 (1.0003, 1.0014)	0.001	1.0007 (1.0001, 1.0014)	0.026	1.0011 (1.0003, 1.0018)	0.005
CBD diameter on US	1.1327 (1.0758, 1.1927)	<0.001	1.1431 (1.0339, 1.2638)	0.011	1.1233 (1.0575, 1.1932)	<0.001
Gallbladder sludge on US	0.7987 (0.5078, 1.2562)	0.322	1.1599 (0.5523, 2.4360)	0.699	0.6857 (0.3848, 1.2219)	0.188
Cholecystitis on US	1.5713 (1.1363, 2.1728)	0.007	2.6537 (1.4698, 4.7911)	0.002	1.1997 (0.8104, 1.7758)	0.365

OR: odds ratio; CBD: common bile duct; US: ultrasound; Values in parentheses: 95% confidence intervals

**Table 4:** Results of logistic binary regression analysis of combined LFT and US results (independent variable) and CBD stone (dependent variable)

	All patients			Age <55			Age ≥55		
	No	OR	P-value	No	OR	P-value	No	OR	P-value
Normal liver enzymes + Normal CBD	138	0.28 (0.146, 0.55)	<0.001	64	NS	NS	74	0.46 (0.23, 0.91)	0.016
Normal liver enzymes + Dilated CBD	106	0.14(0.05, 0.39)	<0.001	50	0.25 (0.06, 1.04)	0.02	56	0.10 (0.03, 0.43)	<0.001
Isolated rise of liver enzymes + Normal CBD	190	0.23(0.12, 0.42)	<0.001	86	0.43 (0.18, 1.04)	0.04	104	0.1587(0.068, 0.37)	<0.001
Isolated rise of liver enzymes + Dilated CBD	90	1.17 (0.69, 1.98)	0.545	40	1.75 (0.76, 4.03)	0.208	50	0.9938 (0.51, 1.96)	0.986
Hyperbilirubinemia + Normal CBD	346	1.52 (1.11, 2.08)	0.01	120	1.23 (0.67, 2.28)	0.502	226	1.54 (1.06, 2.24)	0.025
Hyperbilirubinemia + Dilated CBD	152	5.12 (3.54, 7.41)	<0.001	48	6.85 (3.49, 13.43)	<0.001	104	4.31(2.77, 6.72)	<0.001

LFT: liver function tests; CBD: common bile duct; US: ultrasound; MRCP: magnetic resonance cholangiopancreatography; OR: odds ratio; Values in parentheses: 95% confidence intervals; NS: Not estimable

**Outcomes**

We considered frequency of CBD stones in each group as primary outcome measure. The MRCP report validated and confirmed by a consultant radiologist was considered as evidence for presence or absence of CBD stone. A second independent consultant radiologist who was blinded to original MRCP report reviewed the images to confirm presence or absence of CBD stones. An independent third consultant radiologist was consulted in the event of disagreement. The secondary outcome measures included: predictors of CBD stone, and negative and positive predictive values of combined LFTs and US in detection of CBD stone.

**Data** **collection**

A comprehensive data collection proforma was created for data collection. The data collection proforma included patients’ demographic data (age and sex), diagnoses (biliary colic and cholecystitis), LFTs (bilirubin, ALP, ALT), US scan results (gallbladder stone, gallbladder sludge, CBD stone, cholecystitis, CBD diameter) and MRCP results (CBD stone, CBD diameter, time-interval in days from admission to MRCP). For each patient, the aforementioned data were extracted independently by two authors. Any discrepancies was resolved by discussion between the authors. An independent third author was consulted in the event of disagreement. 

**Data analysis**

In current literature, the incidence of CBD stones ranges from 6% to 38% in patients with normal liver enzymes and normal CBD, 38% to 61% in patients with liver enzymes and dilated CBD; 28% to 50% in patients with abnormal liver enzymes and normal CBD; and 40% to 72% in patients with abnormal liver enzymes and dilated CBD (6,7). We hypothesized that the incidence of CBD stones in the population of this study would be 20% in Group A, 50% in Group B, 40% in Group C, 60% in Group D, 60% in group E, and 70% in group F. Therefore, in order to achieve 80% power with 95% confidence level, it was estimated that a minimum total number of 613 patients would be required: 116 in Group A, 101 in Group B, 116 in Group C, 48 in Group D, 48 in Group E, and 184 in Group F. 

We constructed binary logistic regression models to investigate whether normal or abnormal liver enzymes combined with US scan results can predict the presence of CBD stones. We considered presence of CBD stones detected by MRCP as dependent variable and the following variables as independent variables: 1) Normal liver enzymes with normal CBD diameter on US scan, 2) Normal liver enzymes with dilated CBD, 3) Isolated rise of liver enzymes with normal CBD diameter, 4) Isolated rise of liver enzymes with dilated CBD, 5) Hyperbilirubinemia with normal CBD diameter, and 6) Hyperbilirubinemia with dilated CBD. A two-sided confidence interval (CI) with 95% confidence level was used to indicate statistical significance. Statistical analyses was performed using Minitab 17 (Minitab® 17.1.0). 

## Results

**Baseline characteristics**

Between January 2014 and January 2017, 1423 patients underwent MRCP for suspected CBD stone of which 1022 patients were eligible for this study. Among these, 138 patients had normal liver enzymes with normal CBD diameter on US; 106 had normal liver enzymes with dilated CBD; 190 had isolated rise of liver enzymes with normal CBD diameter; 90 had isolated rise of liver enzymes with dilated CBD; 346 had hyperbilirubinemia with normal CBD diameter; 152 had hyperbilirubinemia with dilated CBD.

Overall, the mean age of included population was 58.6 (SD: 0.59); 39.9% of patients were aged <55 and 60.1% were aged ≥ 55. In terms of gender, 66.3% of patients were female and 33.7% were male. In terms of baseline LFTs, mean value for bilirubin level was 32.5 µmol/L (SD: 1.06); ALP was 199.2 IU/L (SD: 5.19); ALT was 180.8 IU/L (SD: 8.74). The mean diameter of CBD on US scan was 7.2 mm (SD: 0.09); 15.1% of patients were found to have gallbladder sludge on US and 29.3% of patients had diagnosis of acute cholecystitis on US scan. The median time-interval from clinical suspicion of CBD stone and MRCP was 8 days (IQR: 4, 21). The baseline characteristics of included population based on LFTs and CBD dimeters are presented in Table 1.

**Frequency of CBD stone**

Overall, the frequency of CBD stone in the entire cohort was 19.7% (95% CI 17.4%-22.4%). The frequency of CBD stone was 7.2% (95% CI 3.7%-13.3%) in patients with normal liver enzymes and normal CBD diameter; 3.8% (95% CI 1.2%-9.9%) in patients with normal liver enzymes and dilated CBD; 6.3% (95% CI3.4% -11.0%) in patients with isolated rise of liver enzymes and normal CBD diameter; 22% (95% CI 14.4%-32.4%) in patients with isolated rise of liver enzymes and dilated CBD; 24.2% (95% CI 19.9%-29.2%) in patients with hyperbilirubinemia and normal CBD diameter; 47.4% (95% CI 39.3%-55.6%) in patients with hyperbilirubinemia and dilated CBD (Table 2).

Among those aged <55 years old, the frequency of CBD stone in the entire cohort was 13.2% (95% CI 10.2%-17.0%). The frequency of CBD stone was 0% in patients with normal liver enzymes and normal CBD diameter; 4% (95% CI 0.7% -14.9%) in patients with normal liver enzymes and dilated CBD; 6.9% (95% CI 2.8%-15.1%) in patients with isolated rise of liver enzymes and normal CBD diameter; 20% (95% CI 9.6%-36.1%) in patients with isolated rise of liver enzymes and dilated CBD; 15% (95% CI 9.4%-22.9%) in patients with hyperbilirubinemia and normal CBD diameter; 41.7% (95% CI 27.9%-56.7%) in patients with hyperbilirubinemia and dilated CBD (Table 2).

Among those aged ≥ 55 years old, the frequency of CBD stone in the entire cohort was 24.1% (95% CI 20.8%-27.7%). The frequency of CBD stone was 13.5% (95% CI 7.0%-23.9%) in patients with normal liver enzymes and normal CBD diameter; 3.6% (95% CI 0.6% -13.4%) in patients with normal liver enzymes and dilated CBD; 5.8% (95% CI 2.4%-12.6%) in patients with isolated rise of liver enzymes and normal CBD diameter; 24% (95% CI 13.5%-38.4%) in patients with isolated rise of liver enzymes and dilated CBD; 29.2% (95% CI 23.5%-35.7%) in patients with hyperbilirubinemia and normal CBD diameter; 50% (95% CI 40.1%-59.9%) in patients hyperbilirubinemia and dilated CBD (Table 2).

Among those without cholecystitis on US, the frequency of CBD stone in the entire cohort was 17.6% (95% CI 14.9%-20.6%). The frequency of CBD stone was 6.6% (95% CI 3.1%-12.9%) in patients with normal liver enzymes and normal CBD diameter; 0% in patients with normal liver enzymes and dilated CBD; 4.6% (95% CI 1.9%-10.2%) in patients with isolated rise of liver enzymes and normal CBD diameter; 27.6% (95% CI 17.1%-41.1%) in patients with isolated rise of liver enzymes and dilated CBD; 25.4% (95% CI 20.1%-31.6%) in patients with hyperbilirubinemia and normal CBD diameter; 37.4% (95% CI 28.0%-47.7%) in patients with hyperbilirubinemia and dilated CBD (Table 2).

Among those with cholecystitis on US, the frequency of CBD stone in the entire cohort was 25.1% (95% CI 20.4%-30.5%). The frequency of CBD stone was 12.5% (95% CI 2.2%-39.6%) in patients with normal liver enzymes and normal CBD diameter; 14.3% (95% CI 4.7%-33.6%) in patients with normal liver enzymes and dilated CBD; 10% (95% CI 4.1%-21.2%) in patients with isolated rise of liver enzymes and normal CBD diameter; 12.5% (95% CI 4.1%-29.9%) in patients with isolated rise of liver enzymes and dilated CBD; 21.9% (95% CI 14.7%-30.9%) in patients with hyperbilirubinemia and normal CBD diameter; 66.0% (95% CI 51.6%-78.1%) in patients with hyperbilirubinemia and dilated CBD (Table 2).

**Predictors of CBD stones**

*Individual baseline variables*

 The following individual variables were independent predictors of CBD stone: age (OR: 1.0251, 95% CI: 1.0160, 1.0344, P <0.001), gender (OR: 1.4553, 95% CI: 1.0601, 1.9979, P=0.021), bilirubin (OR: 1.0131 95% CI: 1.0090, 1.0172, P <0.001), ALP (OR: 1.0037, 95% CI: 1.0028, 1.0047, P<0.001), ALT (OR: 1.0009, 95% CI: 1.0003, 1.0014, P=0.001), CBD diameter on US (OR: 1.1327, 95% CI: 1.0758, 1.1927, P<0.001), and cholecystitis on US (OR: 1.5713, 95% CI: 1.1363, 2.1728), P=0.007) (Table 3).

*Normal liver enzymes with normal CBD diameter* Binary logistic regression analysis showed that normal liver enzymes with normal CBD diameter does not predict the presence of CBD stone (OR: 0.28, 95% CI 0.146, 0.55, P <0.001) (Table 4).. The results were consistent in patients aged <55 (OR: not estimable), patients aged ≥ 55 (OR: 0.46, 95% CI: 0.23, 0.91, P=0.016), patients without cholecystitis (OR: 0.28, 95% CI: 0.13, 0.59, P<0.001), and patients with cholecystitis (OR: 0.41, 95% CI: 0.09, 1.85, P=0.201).

*Normal liver enzymes with dilated CBD*

Binary logistic regression analysis showed that normal liver enzymes with dilated CBD does not predict the presence of CBD stone (OR: 0.14, 95% CI 0.05, 0.39, P <0.001) (Table 4). The results were consistent in patients aged <55 (OR: 0.25, 95% CI: 0.06, 1.04, P=0.02), patients aged ≥ 55 (OR: 0.10, 95% CI: 0.03, 0.43, P<0.001), patients without cholecystitis (OR: 0.17, 95% CI: 0.03, 0.44, P<0.001), and patients with cholecystitis (OR: 0.47, 95% CI: 0.16, 1.40, P=0.144).

*Isolated rise of liver enzymes with normal CBD diameter*

Binary logistic regression analysis showed that isolated rise of liver enzymes with normal CBD diameter does not predict the presence of CBD stone (OR: 0.23, 95% CI 0.12, 0.42, P <0.001) (Table 4). The results were consistent in patients aged <55 (OR: 0.43, 95% CI: 0.18, 1.04, P=0.04), patients aged ≥ 55 (OR: 0.16, 95% CI: 0.068, 0.370, P<0.001), patients without cholecystitis (OR: 0.23, 95% CI: 0.12, 0.42, P<0.001), and patients with cholecystitis (OR: 0.27, 95% CI: 0.11, 0.67, P=0.001).

*Isolated rise of liver enzymes with dilated CBD*

Binary logistic regression analysis showed that isolated rise of liver enzymes with dilated CBD does not predict the presence of CBD stone (OR: 1.17, 95% CI: 0.69, 1.98, P=0.545) (Table 4). The results were consistent in patients aged <55 (OR: 1.75, 95% CI: 0.76, 4.03, P=0.208), patients aged ≥ 55 (OR: 0.99, 95% CI: 0.51, 1.96, P=0.986), patients without cholecystitis (OR: 1.90, 95% CI: 1.03, 3.5, P=0.048), and patients with cholecystitis (OR: 0.39, 95% CI: 0.13, 1.16, P=0.064).

*Hyperbilirubinemia with normal CBD diameter*

Binary logistic regression analysis showed that hyperbilirubinemia with normal CBD diameter predicts the presence of CBD stone (OR: 1.52, 95% CI: 1.11, 2.08, P=0.010) (Table 4). It did not predict the presence of CBD stone in patients aged <55 (OR: 1.23, 95% CI: 0.67, 2.28, P=0.502) and patients with cholecystitis (OR: 0.76, 95% CI: 0.43, 1.31, P=0.317) but it predicted the presence of CBD stone in patients aged ≥ 55 (OR: 1.54, 95% CI: 1.06, 2.24, P= 0.025) and patients without cholecystitis (OR: 1.52, 95% CI: 1.11, 2.08, P<0.001).

*Hyperbilirubinemia with dilated CBD*

Binary logistic regression analysis showed that hyperbilirubinemia with dilated CBD predicts the presence of CBD stone (OR: 5.12, 95% CI: 3.54, 7.41, P<0.001) (Table 4). The results were consistent in patients aged <55 (OR: 6.85, 95% CI: 3.49, 13.43, P<0.001), patients aged ≥ 55 (OR: 4.31, 95% CI 2.77, 6.72, P<0.001), patients without cholecystitis (OR: 5.12, 95% CI 2.22, 5.63, P<0.001), and patients with cholecystitis (OR: 10.01, 95% CI: 5.17, 19.41, P>0.001).

**Predictive value**

*Positive predictive value (PPV)*

Hyperbilirubinemia with dilated CBD had PPV of 47.37% (95% CI 41.06-53.76); hyperbilirubinemia with normal CBD diameter had PPV of 24.28% ( 95% CI 22.44-26.22); isolated rise of liver enzymes with dilated CBD had PPV of 22.22% (95% CI 15.25-31.20); isolated rise of liver enzymes with normal CBD diameter had PPV of 6.32% (95% CI 4.23-9.32).

*Negative predictive value (NPV)*

Hyperbilirubinemia with dilated CBD had NPV of 94.25% (95% CI 92.98-95.31); hyperbilirubinemia with normal CBD diameter had NPV of 100%; isolated rise of liver enzymes with dilated CBD had NPV of 87.98% (95% CI 87.17-88.75); isolated rise of liver enzymes with normal CBD diameter had NPV of 98.56% (95% CI 97.86-99.03).

## Discussion

We performed a retrospective study to determine whether combined LFTs and US scan results can be used to identify a subgroup of patients with suspected CBD stone who should be selected for biliary tract imaging and those should be selected for direct ERCP. The results of the analyses suggested that the frequency of CBD stone was 7.2% in patients with normal liver enzymes and normal CBD diameter; 3.8% in patients with liver enzymes and dilated CBD; 6.3% in patients with isolated rise of liver enzymes and normal CBD diameter; 22% in patients with isolated rise of liver enzymes and dilated CBD; 24.2% in patients with hyperbilirubinemia and normal CBD diameter; 47.4% in patients with hyperbilirubinemia and dilated CBD. Moreover, we found that normal or isolated rise of liver enzymes (normal bilirubin) with or without dilated CBD on US do not predict the presence of CBD stone whereas hyperbilirubinemia with or without dilated CBD on US predicts the presence of CBD stone. However, combined LFTs and US results had suboptimal PPV for detection of CBD stone with acceptable NPV.

Combining LFTs with ultrasound results to predict CBD stone has been investigated in the past. Barkun and colleagues (8) suggested a model to predict the probability of CBD stone based on combined LFTs and US results. The probabilities reported by Barkun and colleagues8 are significantly greater that those found in our study. Barkun and colleagues (8) used ERCP to detect CBD stone while we used MRCP to evidence the presence of CBD stones. Although ERCP is more accurate than MRCP in detection of CBD stones, this does not explain such a difference between our findings and findings reported by Barkun and colleagues. Considering that the sample size in study by Barkun et al was small, we believe that the probabilities of CBD stones for various subgroups were overestimated in their study. The reported probabilities for patients aged > 55 was significantly higher than probabilities for those aged< 55 in study by Barkun and colleagues.8 Although we found that the age of patient is an independent predictor of CBD stones, we did not find significant differences in the probability of CBD stones between the two age groups.

Individual biochemical or ultrasound findings had been shown to be independent predictors of CBD stones 9, 10. Consistent with previous studies, we found that individual variables such as age, gender, bilirubin, ALP, ALT, CBD diameter on US and cholecystitis on US are predictors of CBD stones. Nevertheless, individual demographic, biochemical, or US scan findings should not be used in isolation to predict CBD stones (7).

Appropriate selection of patients with suspected CBD stone for further imaging (MRCP or EUS) or for ERCP used to be a subject of debate. The British Society of Gastroenterology (BSG) recommended that ERCP should not be used solely as a diagnostic test in patients with suspected CBD stone and should be reserved for patients in whom the clinician is confident an intervention will be required (7). BSG recommended that MRCP and EUS are highly effective in detection of CBD stones in patients that are suspected to have CBD stones (7).

The universal preoperative application of MRCP for detection of CBD stones in patients with suspected CBD stone has been criticized. Epelboym et al. (4) argued that although MRCP is highly effective in the diagnosis of CBD stones, universal application is not cost-effective due to the added overall cost of treatment in terms of time spent in the hospital and delay in definitive management.4 Furthermore, a significant proportion of patients undergoing MRCP do not have CBD stones or might have passed stones (4).

The results of current study may have some implications in terms of appropriate patient selection for further imaging or endoscopic treatment of CBD stones. Based on our results and the best available evidence in the current literature, it could be argued that patients with symptomatic gallstone disease who have normal liver enzymes with or without dilated CBD on US scan should not routinely undergo biliary tract imaging solely for detection of CBD stone. Patients with isolated rise of liver enzymes or hyperbilirubinemia with or without dilated CBD on US scan should undergo biliary imaging prior to ERCP considering the suboptimal PPV of combined LFTs and US.

To the best of our knowledge the current study is the first study in the current literature with adequate statistical power that provides evidence on predictive significance of combined LFTs and US scan findings in detection of CBD stones in patients with symptomatic gallstone disease. We tried to minimize the risk of observer bias, measurement bias and detection bias by enrolling at least two independent authors for data collection, data analysis and interpretation. The reported outcomes of our review should be viewed and interpreted in the context of inherent limitations. The current study was a retrospective study that was conducted in a single center, inevitably subjecting our results to potential selection bias. We used MRCP for detection of CBD stones which is not the gold standard diagnostic tool for detection of CBD stones; therefore, the probability of CBD stones might have potentially been underestimated in our study. However, considering the differences in sensitivity and specificity of MRCP, ERCP and intraoperative cholangiogram in detection of CBD stones, we do not believe that this potential underestimation of probabilities would affect the overall interpretation of our results.

Based on the results of current study and the best available evidence in the literature, it could be argued that patients with symptomatic gallstone disease who have normal liver enzymes with or without dilated CBD on US scan should not routinely undergo biliary tract imaging solely for detection of CBD stone. Patients with isolated rise of liver enzymes or hyperbilirubinemia with or without dilated CBD on US scan should undergo biliary tract imaging prior to ERCP as the PPV of combined LFTs and US does not justify direct ERCP. Direct ERCP could be preserved for patients with high suspicion of CBD stone (e.g. hyperbilirubinemia or dilated CBD on US) in whom clinical features do not justify waiting for biliary tract imaging.

## Conflict of interests

The authors declare that they have no conflict of interest.
